# Cross-sectional analysis of wound-associated soluble factors in early, established, and chronic wounds of recessive dystrophic epidermolysis bullosa patients

**DOI:** 10.1007/s00403-025-04293-w

**Published:** 2025-06-04

**Authors:** Vitali Alexeev, Leonie Huitema, Taylor Phillips, Paras Patel, Mauricio Salas Garza, Franziska Ringpfeil, Julio Cesar Salas-Alanis, Olga Igoucheva

**Affiliations:** 1 Department of Medical Oncology, Thomas Jefferson University, 1025 Walnut Street, Suite 1003, Philadelphia, PA 19107 USA; 2 Department of Dermatology and Cutaneous Biology, Sidney Kimmel Medical College, Thomas Jefferson University, 233 South 10th Street, BLSB, Suite 430, Philadelphia, PA 19107 USA; 3 Rowan University School of Osteopathic Medicine, NJ Rowan, USA; 4 Institute de Tecnologico de Monterrey, Monterrey, NL Mexico; 5 Ringpfeil Advanced Dermatology, Philadelphia, PA USA; 6 Universidad Autonoma de Nuevo Leon, Monterrey, NL Mexico

**Keywords:** RDEB, Wound healing, Chronic wounds, Inflammation, Growth factors, Cytokines, Chemokines, Angiogenesis

## Abstract

**Background:**

Poorly healing wounds represent the primary health-related burden for hereditary recessive dystrophic epidermolysis bullosa (RDEB) patients. Contribution of wound-associated soluble constituents to wound progression remains not well defined.

**Objective:**

To conduct cross-sectional analysis of cytokine, chemokine, and growth factor in exudates from RDEB wounds and define changes associated with wound progression.

**Methods:**

Concentrations of selected cytokines, chemokines, and growth factors were evaluated by multiplex ELISA in eight blister fluids and 66 exudates from early, established, and chronic RDEB and five chronic venous ulcers (VU). A cross-sectional analysis was performed.

**Results:**

Our data demonstrated that proinflammatory CXCL8 and IL-1β tend to accumulate in established RDEB lesions. The levels of several interleukins including IL-17, IL-18, and IL-10 were significantly higher in RDEB chronic wounds than in VU. Contrary to VU and other chronic wounds, high levels of VEGF, G-CSF, and HGF growth factors were detected in RDEB established and chronic skin lesions.

**Conclusion:**

Although this study is limited to cross-sectional analysis of wound exudates, detected high levels of specific pro-inflammatory, neutrophil-recruiting, and pro-angiogenic and pro-proliferative factors, such as IL-1β, CXCL8, VEGF, G-CSF, and HGF define RDEB wounds and offer potential pharmacological targets to improve wound healing in the patients.

**Supplementary Information:**

The online version contains supplementary material available at 10.1007/s00403-025-04293-w.

## Introduction

Hereditary recessive dystrophic epidermolysis bullosa (RDEB) is a mechanobullous skin fragility disorder with considerable morbidity and mortality [1]. RDEB is caused by mutations in *COL7A1* gene that lead to lack or dysfunction of type VII collagen (Col7) at the dermal-epidermal junction and extreme fragility of the skin. It manifests in separation of skin layers following minor mechanical stress to the skin and development of skin lesions ranging from blisters to chronic ulcerated wounds. The latter, being associated with infection, dehydration, deformities, and cancer [2, 3] presents the primary health-related burden to RDEB patients. The molecular and cellular events outlining or regulating progression of RDEB wounds to chronic state remain not well defined. To delineate mechanisms that can impair cutaneous wound healing in RDEB patients, we have developed a non-invasive sampling method to isolate wound-associated cellular and soluble constituents [4]. Our prior analysis of changes in the cellular constituents showed that RDEB wound progression is associated with continuous recruitment of mature neutrophils to the wounds, reduced number of macrophages, and the presence of activated antigen-presenting and T cells [4]. Here, we defined and quantified 13 of the most common cytokines, chemokines, and growth factors associated with different stages of RDEB wound progression using multiplex ELISA assay and outlined putative molecular mechanisms that could impair wound healing in RDEB patients.

## Matherials and methods

### Subjects and samples

Patients or their legal guardians received counseling pertaining to the study and gave written informed consent. Eight blister fluids from early blisters (1–2 days old; separated epidermis, blister cup and blister fluid are present) and 66 exudates from early (1–7 days old; open wound, no blister cup present; *n* = 35), established (1–3 weeks old; open wounds; no signs of re-epithelization; *n* = 14) and poorly healing/chronic (> 3 weeks up to 2 years old; open wounds, no re-epithelization; *n* = 17) wounds and venous ulcers (VU). VU samples were collected from chronic skin lesions (more than 4 weeks old, open wounds; no re-epithelization). The study was reviewed and approved by the local IRB. A total of 80 samples were collected from 76 RDEB patients. Several samples were excluded from the analyses due to low quantity or contamination. Distribution of samples by wound stage, patient age, gender, and location on the body is presented in the supplementary materials.

### Collection wound-covering bandages and recovery of soluble bandage-derived constituents

Detailed protocol for recovery of wound exudates was provided previously. ^4^ Briefly, non-occlusive wound dressings (~ 20 × 20 mm^2^) were collected from the center of a wound and placed in transport media on ice. Bandages containing ointments were excluded. All wounds had no documented active bacterial infections. Within 24 h, wound dressings were scraped and removed from the transport media. The transport media was centrifuged at 200xg for 10 min. Supernatants were clarified by sequential filtration on 1.5 μm (Environmental Express) and 0.22 μm filters (Millipore), respectively. High molecular weight protein fraction was collected by centrifugation on Ultracel-100 K concentrators (Millipore). The flow through was further concentrated on Ultracel- 3 K (Millipore) units to obtain ~ 250 µl of low molecular weight (LMW) fraction. Protein concentration in fractions was measured using 660 reagent (Themo-Fisher) and normalized by human serum albumin measured by ELISA (Abcam). All procedures were performed at 4^0^ C. A total of 76 RDEB-derived exudates were collected. Ten exudates were excluded from the analyses due to contamination, and 66 samples were analyzed.

### LEGENDplex^tm^ multi-analyte ELISA

Soluble constituents of the wounds were assessed by human inflammatory chemokines (13-plex), human inflammation (13-plex), and human growth factors (13-plex) LEGENDplex^tm^ bead-based multiplex panels according to the manufacturer’s protocol (BioLegend, San Diego, CA). Data were acquired using a Guava EasyCyte System and analyzed using GuavaSoft 2.7 software (Millipore). Curve fitting of the standards’ mean fluorescent intensities and quantitative calculation of the samples’ mean fluorescent intensities to pg/ml were achieved utilizing LEGENDplex^tm^ software.

### Statistical analysis

Jarque-Bera test was used to ensure that the values in the obtained dataset are normally distributed.

Comparison of the data was performed using two-way repeated measures ANOVA test and Student’s 2-tailed *t*-test. A p-value of < 0.05 was considered statistically significant. R-squared test was used to determine whether variance of the soluble factors in the exudates was defined by the wound type.

## Results

### Pro-inflammatory chemokines were abundant in early and established RDEB wounds

**Fig. 1:**
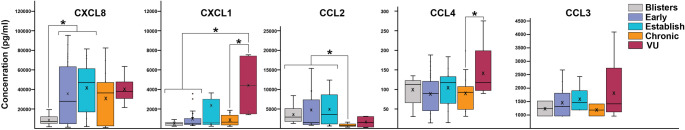
Analysis of RDEB wound-associated chemokines. Multiplex analysis of deregulated chemokines most commonly present in RDEB skin-wound exudates (indicated above the charts). Exudates from chronic venous ulcers (VUs) were used to establish differences between chronic RDEB and VU wounds. Data are presented as concentration (pg/ml) in box and whisker plots displaying variations in specific chemokines and allowing comparison between different RDEB wound types (color-coded as shown in the key). X and bar on boxes show mean and median values, respectively. Outliers are shown as dots. Asterisk indicates statistically significant differences (*p* < 0.05) between specific wound types

Immediately after wounding, wound-associated cells secrete chemokines that recruit specific leukocytes and stromal cells to stimulate wound healing [5]. To define chemotactic signals that could mediate aberrant recruitment of leukocytes to RDEB skin wounds [4], we analyzed 13 most common wound-bed-associated pro-inflammatory chemokines (CCL2, CCL3, CCL4, CCL5, CCL11, CCL17, CCL20, CXCL1, CXCL5, CXCL8, CXCL9, CXCL10, CXCL11) in wound exudates. Of all examined chemokines, CXCL8 was detected at the highest concentrations. Its concentrations were lower (7 ng/ml)^1^ in blister fluids (BF) and higher in early (28 ng/ml) and established (49 ng/ml) RDEB wounds. CXCL8 levels were somewhat lower (38 ng/ml) in chronic lesions and were compatible with VU (39 ng/ml) (Fig. 1). Based on the high value of the coefficient of determination (R^2^ = 0.99) nearly 100% of examined RDEB samples followed this trend (Fig. 4). Somewhat similar trends showing accumulation of CXC1 in established lesions were detected in RDEB (Figs. 1 and 4). Significant changes in CCL3 and CCL4 levels were not detected in RDEB, although these chemokines were present at higher levels in VU (Fig. 1). CCL20 (600 pg/ml), CXCL5 (250 pg/ml) and CXCL10 (2.2ng/ml) were significantly higher in RDEB BF than in exudates (Fig, S1). Other measured chemokines were either not detected in RDEB (CXCL11, CCL17), or detected only in a few RDEB samples (CCL5, CXCL9) (data not shown).

### Pro-inflammatory IL-1β, IL-6, IL-17, and IL-18 dominated RDEB wound exudates

**Fig. 2:**
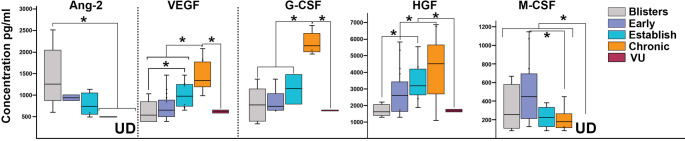
Analysis of RDEB wound-associated cytokines. (**a**) Multiplex analysis of deregulated cytokines most commonly present in RDEB skin-wound exudates (indicated above the charts). Exudates from chronic venous ulcers (VUs) were used to establish differences between chronic RDEB and VU wounds. Data are presented as concentration (pg/ml) in box and whisker plots displaying variations in specific molecules in different RDEB wound types (color-coded as shown in the key). X and bar on boxes show mean and median values, respectively. Asterisk indicates statistically significant differences (*p* < 0.05) between specific wound types. UD– indicates undetected molecules. (**b**) Changes in values of selected cytokines (log-transformed, indicated in the key) in blister fluids (BF) and exudated from early (EA), established (EST), and chronic (CHR) RDEB wounds. (**c**, **d**) Dot plots illustrating Pearson correlation between IL-1β, CXCL8, and CXCL1 in different fluids (indicated in the key). Correlation coefficients are shown in the plots. Trend lines (red) illustrate linear correlation between IL-1β, CXCL8, and CXCL1

Detection of 13 most common inflammatory cytokines by multiplex ELISA showed that seven of them are consistently present in RDEB wound exudates. IL-1β, IL-17, IL-6, IL-10, and IL-18 were present at lower levels in RDEB blister fluids but were substantially higher in early and established wounds (Fig. 2a). Fluctuated levels of IL-6 were detected in all RDEB wounds with lower levels in BF (2.2 ng/ml) and early lesions (1.8 ng/ml) and significantly higher (3.3 ng/ml; *p* < 0.05) levels in established wounds (Fig. 2a). Of all tested cytokines, accumulation of IL-1β at wound sites from undetected in BF to 5.2 ng/ml in early and 17 ng/ml in established RDEB wounds was observed. This cytokine peaked at 30–35 ng/ml in established lesions and were significantly higher (*p* < 0.05) than in VU exudates (3.8 ng/ml) (Fig. 2a). Log transformed data plots showed a substantial increase of IL-1β associated with progression of wounds to established and chronic state (Fig. 2b). Pearson correlation analysis of the log-transformed data showed that wound progression-associated changes in IL-1β correlate extremely well with changes in CXCL8 (*r* = 0.99) and with changes in CXCL1 levels (*r* = 0.79) (Fig. 2c, d).

Along with IL-1β, two pro-inflammatory cytokines, IL-17 and IL-18, were present in all RDEB lesions. IL-17 was detected at low levels in BF and early wounds (~ 45 pg/ml). Its concentration was significantly (*p* < 0.05) increased in established lesions (110 pg/ml). This cytokine was not detectable in VUs. IL-18 levels fluctuated in RDEB wounds with the highest concentrations (340 pg/ml) detected in chronic wounds (Fig. 2). TNFα, another inflammatory cytokine, was detected only in 1/3 of all RDEB lesions and its levels fluctuated from 50 pg/ml to 50 ng/ml independently of the wound type (not shown). IL-10, a known anti-inflammatory cytokine, was also present in all types of RDEB lesions. Its levels were elevated in early (170 ng/ml) and established (120 ng/ml) wounds and were somewhat lower in chronic lesions (77 pg/ml). IL-10 levels in all RDEB wounds were significantly higher than in VUs (*p* < 0.05) (Fig. 2). Trend lines for IL-6, IL-17 and IL-10 changes suggest accumulation of these cytokines during wound progression toward poorly healing state (Fig. 4).

### Growth factors were abundant in RDEB wound exudates

**Fig. 3:**
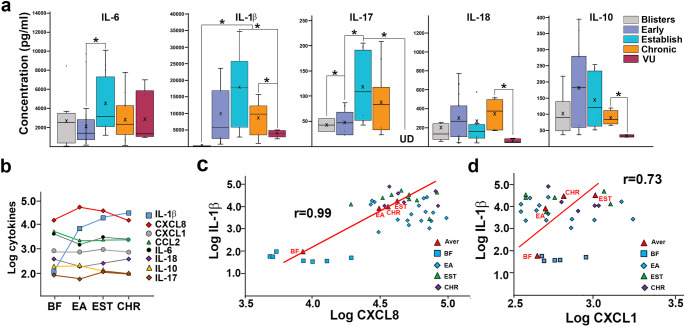
Analysis of RDEB wound-associated growth factors. Multiplex analysis of deregulated growth factors most commonly present in RDEB skin wound exudates (indicated above the charts). Exudates from chronic venous ulcers (VUs) were used to establish differences between chronic RDEB and VU wounds. Data is presented as concentration (pg/ml) in box and whiskers plots displaying variations in specific growth factors and allowing comparison between different RDEB wound types (color-coded as shown in the key). X and bar on boxes show mean and median values, respectively. Outliers are shown as dots. Asterisk indicates statistically significant differences (*p* < 0.05) between specific wound types. UD– indicates undetected molecules

**Fig. 4:**
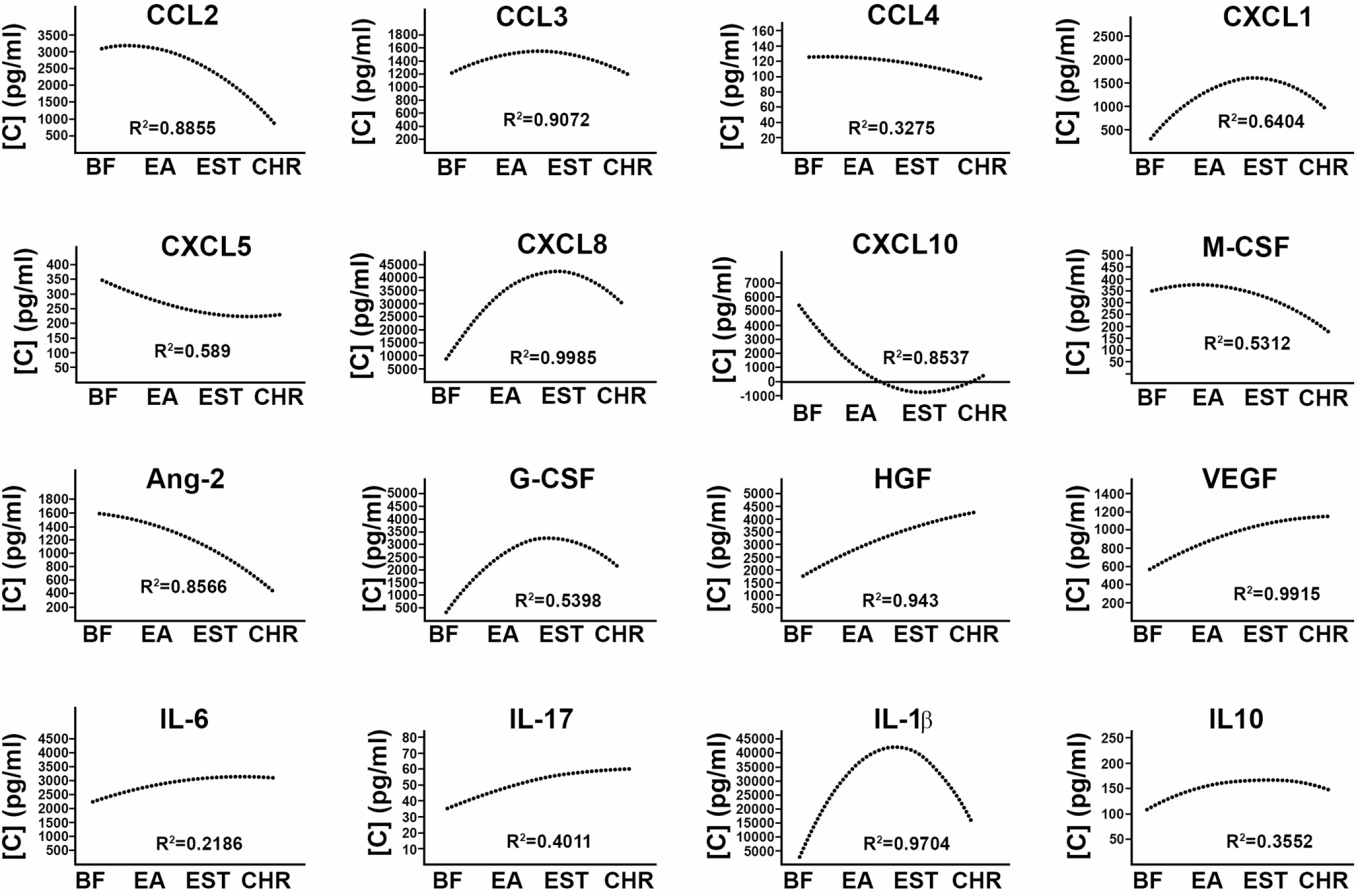
Trend lines depicting changes in wound bed-associated chemokines, growth factors, and cytokines. Trend lines for individual molecules were calculated from the data presented in Figs. 1 and 2, and 3 for individual chemokines, growth factors, and cytokines (shown above). Lines illustrate changes in concentrations of the wound bed-associated molecules (pg/ml). Results of the R2 test are shown below the trend lines. BF– blister fluids, EA– early wounds, EST– established wounds, CHR– chronic wounds

Evaluation of the growth factors showed that Erythropoietin, basic FGF, GM-CSF, PDGF-BB, and SCF were not present in RDEB exudates. Pro-angiogenic angiopoietin 2 (Ang-2) was consistently present in all examined blister fluids in higher concentrations (1.5 ng/ml) but was detected only in a minority (up to 5%) of all other RDEB wounds at much lower concentration (800 pg/ml) (Fig. 3). Consistent with our prior data [4], vascular endothelial growth factor (VEGF) was detected in all RDEB exudates. The highest levels (1.4 ng/ml) of this pro-angiogenic growth factor were associated with chronic wounds, where they were significantly higher than in other RDEB and VU lesions (*p* < 0.05) (Fig. 3). A similar pattern was also detected for granulocyte colony stimulating factor (G-CSF) and hepatocyte growth factor (HGF) (Fig. 3). Although G-CSF was detected in about 50% of examined exudates, in chronic RDEB wounds its levels (2.1 ng/ml) were significantly higher than in other lesions (*p* < 0.001). Progression of RDEB wounds to chronic state was also associated with HGF accumulation at wounded sites with the highest levels (4.3 ng/ml) detected in chronic lesions. Macrophage colony-stimulating factor (M-CSF) was detected in about 50% of RDEB-derived exudates with higher concentrations ranging between 100 pg/ml and 1 ng/ml in blister fluids and early wounds and lower concentrations (200 pg/ml) in established and chronic lesions. All these growth factors were either not detected in VUs or their levels were significantly lower (*p* < 0.05) than in RDEB (Fig. 3). Epidermal growth factor (EGF) and *t*ransforming growth factor alpha *(*TGFα) were detected in less than 10% of examined RDEB samples with and at an average of 500 pg/ml concentration. These growth factors were not detected in VU lesions (not shown). Collectively, HGF (R^2^ = 0.94), VEGF (R^2^ = 0.99), and G-CSF (R^2^ = 0.53) tend to increase along with wound progression, whereas Ang-2 and M-CSF levels were higher in earlier lesions and substantially lower in the established and chronic wounds (Figs. 3 and 4).

## Discussion

Development of poorly healing and chronic wounds remains the primary health-related burden for RDEB patients. Although herpes virus-based gene therapy was recently approved by the FDA for the treatment of RDEB wounds [6], many patients experience discomfort and substantial bacterial infection due to the use of occlusive dressing during the therapy. Better understanding of the mechanisms controlling stalled wound healing in RDEB could guide design and development of novel and safer therapeutics. Previously, we analyzed cytokines and chemokines in blister fluids from epidermolysis bullosa-affected patients and defined cellular constituents in RDEB skin lesions [4, 7]. In this study, we quantified the most common wound-bed-associated cytokines, chemokines, and growth factors in exudates collected at different stages of RDEB wound progression ranging from early blisters to chronic wounds. Increasing nanogram-range CXCL8 concentrations that reach maximum in established lesions (Figs. 1 and 4) suggest that this multifactorial pro-inflammatory and pro-angiogenic chemokine could play a pivotal role in RDEB wound pathology. It may provide constitutive recruitment of neutrophils that hallmark a stalled inflammatory phase and contributes to continuous tissue damage and progression of wounds to chronic state. This notion is consistent with our prior data showing that 80% of wound-associated leukocytes express CXCR1 and CXCR2 receptors for this chemokine and that accumulation of CXCR2^+^ mature neutrophils define chronic RDEB wounds [4]. Although the precise source of CXCL8 in damaged RDEB skin is not yet defined, it is plausible that most of this chemokine is produced by wound-recruited leukocytes under the influence of other wound-associated factors. One such factor known to induce CXCL8 expression is IL-1β. This cytokine activates nuclear factor kappa-light-chain-enhancer of activated B cells (NF-κB) and c-Jun N-terminal kinase 1 (JNK1) signaling pathways to promote transcription of *CXCL8* gene [8]. Prior studies have shown that acute incisional wounding of the skin induces secretion of up to 1 ng/ml of IL-1β at the wound bed and IL-1β-dependent induction of *CXCL8* [9]. Our data showing progressive accumulation of up to 25 ng/ml IL-1β in established RDEB wounds and correlated CXCL8 levels (Fig. Figures 2c and 4) suggest a mechanistic link between these two pro-inflammatory molecules and their crucial role in RDEB wound pathology. Although current studies were conducted on exudates collected from clinically non-infected lesions, it is also plausible that bacteria at wounded sites augmented secretion of these pro-inflammatory molecules [10–12]. This concept is indirectly supported by our data showing elevated levels IL-17 in established and chronic RDEB lesions. In pathological conditions, this cytokine synergizes with IL-1β and enhances CXCL8 secretion [13] through activation of the AP-1 and NF-kB [8, 14]. IL-17 is predominantly produced by Th17-differentiated CD4^+^ T helper cells to direct adaptive immunity toward infections caused by bacteria, fungi, viruses, and parasites. Although our prior studies did not reveal any significant differentiation of the RDEB wound-derived T cells toward Th17 ex vivo in the presence of *Staphylococcus aureus* antigens [15] observing IL-17 and IL-18 in RDEB established lesions point to bacteria-induced T cell differentiation toward Th17 cells at wounded sites. Nevertheless, high levels of IL-10, a known anti-inflammatory cytokine that suppresses IFNγ and GM-CSF synthesis, antigen-presentation, and T cell activity [16] suggest its immune inhibitory role in RDEB lesions. Lack of the above-referenced cytokines in RDEB lesions indirectly supports this notion.

Contrary to other chronic wounds (e.g., VUs, diabetic foot ulcers, etc.), we found that RDEB wounds contained large quantities of different growth factors. Prior studies by others and us demonstrated elevated blood vessel densities in RDEB skin [4, 17]. Our current data showing high levels of Ang-2 in blisters fluids suggest that this factor could sensitize Tie2^+^ endothelial cells by an autocrine mechanism [18] to enhance vascular morphogenesis and stimulate leukocyte rolling on dermal blood vessels. This initial pro-angiogenic stimuli is further enhanced by VEGF, IL-6, and CXCL8 during wound progression, resulting in highly vascularized and inflamed skin in established and chronic wounds. Accumulation of VEGF in RDEB wounds is accompanied by accumulation of the G-CSF and HGF. Although both growth factors enhance wound healing [19–22], their activity may have a detrimental effect in RDEB. It is plausible that G-CSF provides its angiogenic and anti-apoptotic properties, which support vascularization of the skin, counterweight apoptotic death of wound-associated neutrophils, and enhanced survival of keratinocytes at the wound edge. At the same time, HGF recruits neutrophils, monocytes, and mast cells to the wounds [19] and, along with EGF and TGFα [23] that were detected in some RDEB lesions, enhance keratinocyte proliferation. These pro-survival, pro-proliferative signals could create a favorable milieu for uncontrolled proliferation and tumorigenic transformation of the wound edge-associated keratinocytes resulting in the formation of aggressive squamous cell carcinoma, a well-known health-related burden in RDEB patients.

Considering substantial variation in the levels of several cytokines (e.g. IL-1b, IL-17) and chemokines (e.g. CXCL8) in early/established RDEB wound, we tested whether such variability could result from differences in patients’ age. No significant correlation between the levels of the individual molecules in different lesions and patient age was detected (not shown). Nevertheless, our data correlates with prior findings showing accumulation of IL-6, L-1β, CCL2, CXCL1, and CXCL8 in tissues, blister fluids, and exudates of burn wounds [24–26]. However, concentration of these molecules and other measured cytokines, chemokines, and growth factors in burn wounds [24, 26] are up to two orders of magnitude lower than in established RDEB wounds (Fig. 1). Also, our findings suggest that high levels of pro-inflammatory molecules at wounded sites could be ‘spilled over’ into circulation and result in systemic inflammation in RDEB patients. Indirectly, this notion is supported by our prior studies showing high blood vessel density in RDEB-affected skin [4] and the data showing elevated concentrations of several pro-inflammatory cytokines including IL-1β (~ 37 pg/ml), IL-6 (~ 40 pg/ml), IL-10 (~ 30 pg/ml), and CXCL8 (~ 50 pg/ml) in the blood of RDEB patients [27]. It is also supported by recent findings showing that skin could be a possible source of higher levels of the systemic IL-6 [28]. Our data is also in agreement with the recent transcriptomic profiling of six wound-adjacent perilesional skin samples showing dysregulation of the cytokine and chemokine signaling networks in RDEB [29]. Our study has several limitations: it is limited to the evaluation of the most common cytokines, chemokines, and growth factors and does not cover a broad spectrum of wound bed-associated proteome. It is also restricted to a cross-sectional analysis that does not allow tracing time-dependent changes in the composition of the exudates the individual samples. It does not allow side-by-side comparison of RDEB skin wound-associated signatures with wound healing signatures of normal human skin, and it does not address variability in clinical severity among RDEB patients. Nevertheless, presented here approach permitted non-invasive collection of samples from a rather large cohort of RDEB patients and defined molecular signature on protein level.

Presented here data demonstrated the importance of measuring various signaling proteins in the exudates to accurately reflect the wound-bed status. These data identified several distinct molecular signatures of RDEB lesions when compared to chronic venous ulcers. Also, our study suggests that pharmacological targeting of IL-1β/CXCL8-dependent signaling could be tested as an approach to reduce wound-associated pro-inflammatory signals, prevent progression of the wounds to chronic state, and spillage of these molecules into systemic compartment. Although further studies are necessary to define clinical benefits of such strategies, it is plausible that repetitive draining of wound exudates from open RDEB wounds could reduce pro-inflammatory signals and facilitate wound healing in RDEB patients.

## Electronic supplementary material

Below is the link to the electronic supplementary material.

Supplementary Material 1

## Data Availability

No datasets were generated or analysed during the current study.

## Abbreviations

RDEB: Recessive Dystrophic Epidermolysis Bullosa
APC: Antigen presenting cells
LC: Langerhans cells
MMPs: Matrix metalloproteinases
TIMP-1: Tissue inhibitor of metalloproteinase 1
IL: Interleukin
VEGF: Vascular endothelial growth factor
MVD: Micro/vessel density
DFU: Diabetic foot ulcer
FACS: Fluorescence activated cell sorting

## Footnotes

1 Median values of the detected concentrations are presented in parenthesis.
