# Supplementary material for: Cross-sectional analysis of wound-associated soluble factors in early, established, and chronic wounds of recessive dystrophic epidermolysis bullosa patients
Source: Arch Dermatol Res. 2025 Jun 4;317(1):796. doi: 10.1007/s00403-025-04293-w (PMC12137523; doi:10.1007/s00403-025-04293-w)
Supplement: Supplementary file 1 — Supplementary Material 1 [file 403_2025_4293_MOESM1_ESM.docx]

**Supplementary materials**

**Figure S1.**

**
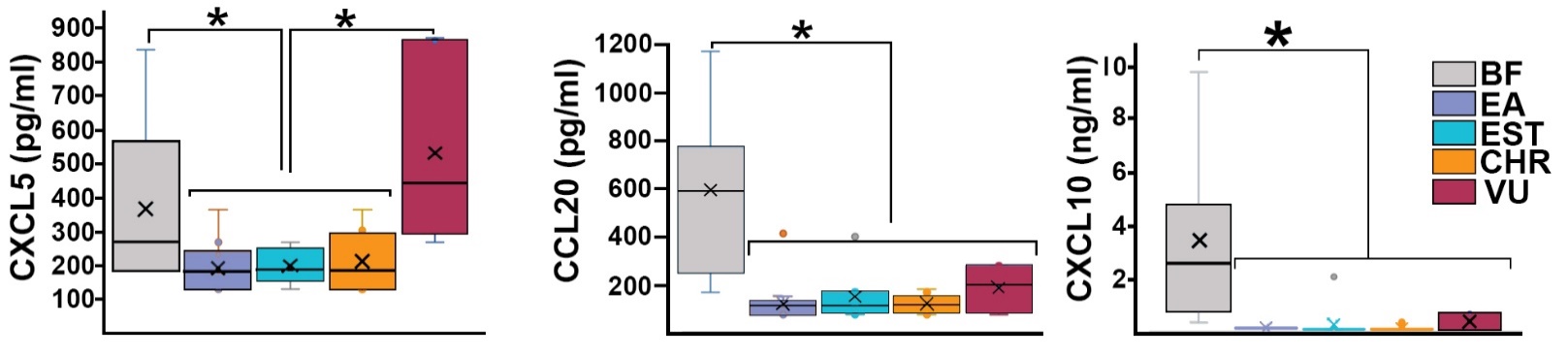
**

Fig.S1 Analysis of CXCL5, CCL20, and CXCL10 (as indicated) in blister fluids (BF) and in exudates from early (EA), establish (EST), chronic (CHR) RDEB wounds, and venous ulcers (VU), as indicated in the key. Data is presented as concentration in box and whiskers plots displaying variations in specific chemokines. X and bar on boxes show mean and median values, respectively. Outliers are shown as dots. Asterisk indicates statistically significant differences (p<0.05) between specific wound types.

**Distribution of samples by wound stage, patient age, gender, and location on the body**

**Distribution of wounds by location and wound stage**:

(location not listed for 1 sample)

**Distribution of wounds by gender:**

(gender not listed for 2 samples, wound stage not listed for 1 sample)

**Distribution of wounds and wound stages by patient age groups:**

(wound stage not listed for 1 sample, patient age not listed for 4 sample)
